# Understanding Intimate Health in Online Spaces Among Women in Aotearoa New Zealand: Protocol for a Mixed Methods Study

**DOI:** 10.2196/65288

**Published:** 2025-12-01

**Authors:** Victoria Egli, Callie Vandewiele, Hanna Price, Caitlyn MacIntyre, Sarah Hendrica Bickerton, Nina Duggan, Seline McNamee, Emmy Rākete, Alana Cavadino

**Affiliations:** 1 Te Huataki Waiora School of Health The University of Waikato Hamilton New Zealand; 2 Department of Anthropology Te Pūtahi Mātauranga | Faculty of Arts and Education Waipapa Taumata Rau | The University of Auckland Auckland New Zealand; 3 Middlemore Hospital Health New Zealand | Te Whatu Ora Auckland New Zealand; 4 Public Policy Institute Te Pūtahi Mātauranga | Faculty of Arts and Education Waipapa Taumata Rau | The University of Auckland Auckland New Zealand; 5 Te Wāhanga Ture | Faculty of Law, and Mātauranga Hauora | Faculty of Medical and Health Sciences Waipapa Taumata Rau | The University of Auckland Auckland New Zealand; 6 Te Puna Mārama | School of Social Sciences Te Pūtahi Mātauranga | Faculty of Arts and Education Waipapa Taumata Rau | The University of Auckland Auckland New Zealand; 7 Epidemiology and Biostatistics Mātauranga Hauora | Faculty of Medical and Health Sciences Waipapa Taumata Rau | The University of Auckland Auckland New Zealand

**Keywords:** sexual and reproductive health, social media, information dissemination, women's health, digital health

## Abstract

**Background:**

Social media has become an important tool facilitating communication and connection in a postpandemic landscape. This context has prompted an increase in the sharing of information and disinformation about general health. This provides an opportunity to seek information about and support for intimate (ie, sexual and reproductive) health, with the added assurance of maintaining a degree of anonymity in a socially taboo context.

**Objective:**

The aim of this study is to better understand how women in Aotearoa New Zealand use social media to access sexual and reproductive health information and support. Engagement with stakeholders and community groups will be undertaken throughout the study to analyze the findings and present solutions to identified issues.

**Methods:**

This online survey is the first stage in a study which uses a mixed methods approach involving the collection of qualitative and quantitative data through an anonymous survey on the Qualtrics platform. Recruitment occurred through advertising on the University of Auckland | Waipapa Taumata Rau and University of Waikato social media accounts and physical posters in various locations nationwide. These posters prioritized recruitment of groups that are often underrepresented. This study will also present the preliminary results to stakeholders, prioritizing *whakawhanaungatanga* (building relationships) and *manaakitanga* (kindness and respect for others, emphasizing responsibility and reciprocity) in the co-designing of future research aims. Subsequent analysis of all the above data forms will be performed using reflexive thematic coding analysis, using codes derived from both the literature and emergent from the data. Results will be disseminated in academic, participant, and stakeholder circles. The analysis plan involves research assistants and investigators working collaboratively and independently, progressively comparing codebooks and results to reiteratively code for the eventual thematic results.

**Results:**

Ethical approval to conduct this study was granted by the University of Auckland ethical review board on June 28, 2023, and an amendment to change survey questions and expand recruitment was approved on July 1, 2024. Online data collection closed in February 2025, and focus groups and interviews are scheduled to begin meeting in August 2025 with stakeholder engagement occurring throughout. Compensation was not provided for completion of the online survey, but will be provided (NZ $50 [US $28.93] vouchers) for focus group and interview participants when stage 3 of the project is undertaken.

**Conclusions:**

From early data analysis, we anticipate survey results will confirm our hypothesis that social media communities can provide increased access to information and support for women who have had or continue to have limited access to intimate health care due to a number of factors, including social (embarrassment), structural (location), or cultural (lack of health care provider training) factors.

**International Registered Report Identifier (IRRID):**

DERR1-10.2196/65288

## Introduction

During the COVID-19 pandemic, social media peaked as a major tool for communicating health information and connecting people to each other during a time of physical distancing; it was widely used by public health professionals and conspiracy theorists alike [1]. Since the beginning of the COVID-19 pandemic, the use of social media for obtaining health information and support on all kinds of health-related topics has increased [2]. The use of social media to convey information and provide support in relation to socially taboo topics, specifically sexual and reproductive health, will be further investigated in this study. We focus on the experiences of women living in Aotearoa New Zealand, and their use of social media for sexual and reproductive health information and support. We define social media as any online environment that builds relationships and facilitates connection as well as the sharing of information and lived experiences of those who use it. Textbox 1 provides definitions of important local terms.

**Local terms of reference**

Hui: a meeting, workshop, or gathering.

Kanohi ki te kanohi: face-to-face interaction; a culturally significant approach in Māori contexts that emphasizes the importance of personal connection, presence, and direct engagement.

Koha: a gift or offering used to acknowledge a contribution, grounded in principles of reciprocity and care for others.

Māori: the Indigenous people of Aotearoa New Zealand.

Manaakitanga: the expression of care, hospitality, and kindness toward others, upholding their dignity and well-being.

Marae: a communal and sacred meeting space that serves as the focal point for Māori social, cultural, and spiritual life, typically including a meeting house (wharenui) and other associated buildings.

Takatāpui: a traditional Māori term that has been reclaimed to describe a person who is Māori and identifies with diverse genders and/or sexualities, encompassing both cultural and LGBTQIA+ identities.

Te reo Māori: the Māori language, an official language of Aotearoa New Zealand and a cornerstone of Māori identity, culture, and heritage.

Whakawhanaungatanga: the process of building and maintaining relationships through shared connections, trust, and mutual respect.

Sexual health is a state of complete physical, emotional, spiritual, mental, and social well-being regarding sexuality, sexual orientation, sexual expression, and gender identity [3]. It also encompasses the ability of a person to have safe and pleasurable sexual experiences [3]. Reproductive health extends this definition and focuses on a complete state of well-being in relation to the body’s reproductive system and processes [3]. Reproductive autonomy is a core feature of this definition, as people must have autonomy over whether, when, and by what means they have a child [3]. The achievement of optimal sexual and reproductive health is dependent on environments that affirm, promote, and respect health for all individuals regardless of their sex assigned at birth or their gender [3]. This includes environments that allow individuals timely access to comprehensive, high-quality, and unbiased information, where they are free from coercion and discrimination [3].

The COVID-19 pandemic accentuated the crucial role of social media in information dissemination. Social media can be an effective communication tool in times of crisis, as the need to quickly disseminate information is particularly pressing [4]. Equally, the interactive nature of social media allows individuals to seek support from others. Social media provides opportunities for connection, identity formation, and a sense of group belonging [5,6]. Online communities on social media provide resources for individuals in relation to sexual and reproductive health information and support [7]—topics that are often shrouded in embarrassment and shame [8].

Despite appearing on the world stage to be socially progressive in many areas [9], Aotearoa New Zealand has a sexually conservative culture [10,11]. This makes seeking sexual and reproductive health information and support difficult for many. Fear of being judged, feeling shy, embarrassed, or ashamed, not knowing how to access care, and concerns about confidentiality are reported as reasons for unmet needs in sexual and reproductive health care service provision in Aotearoa New Zealand [12]. Adolescents and young adults appear to be the demographic most negatively impacted [12,13]. For Māori, stigma and negative societal attitudes toward sexual health intersect with the systemic racism inherent in Aotearoa New Zealand’s health system [14,15]. All of these combine to negatively impact the way sexual and reproductive health information and support are accessed, as well as the quality of formal health care services provided [12,16]. This stigma intersects with other access barriers, such as cost, transport, and cultural fluency, limiting access to intimate health care. We posit that social media and online communities expand access to intimate health care by providing people with anonymous spaces to discuss and share information that they may not be able to discuss and share within their own communities, or in spaces where they lack anonymity.

In Aotearoa New Zealand, people who are Māori or Pasifika, have a disability, are overweight, or identify as lesbian, gay, bisexual, transgender, *takatāpui*, queer/questioning, intersex, asexual/aromantic/agender, or another marginalized identity (LGBTTQIA+) are more likely to have negative, hostile, and stigmatizing interactions with health care services [17-20]. Moreover, women from these vulnerable populations are more likely to face disparities and inequalities due to multiple levels of existing social injustice [21].

Women’s sexual and reproductive health in Aotearoa New Zealand is of particular relevance due to the high incidence of sexually transmitted infections (STIs) in comparison to other Western countries [10,11]. National STI rates in Australia, the United States, and the United Kingdom are lower than regional STI rates in Aotearoa New Zealand [10]. Women in sexual relationships with male partners older than themselves may be particularly at risk of contracting an STI [11]. Other intimate health concerns include the long-term mental and physical impacts of untreated STIs, such as infertility and ectopic pregnancy [12,22], and the incidence of cervical cancer, which is often not adequately screened for in Aotearoa New Zealand [23].

For this study, we have chosen to define women as those who were assigned female at birth, and/or anyone who has ever self-identified or currently self-identifies as a woman. Upon consultation with extended networks, we acknowledge this inclusive definition will not be favored by everyone [24]. Defining gender and sexuality is an active space, and we recognize that these concepts are ever-evolving and that descriptions can and do change over time. Grounded in the underlying philosophical paradigm of intersectional feminism [25], this study’s research question is as follows: In the context of women’s experiences of the Aotearoa New Zealand health system, how do women use social media to find information and support around sexual and reproductive health?

Further, we ask what advantages and disadvantages they perceive regarding such social media use. The findings from pilot testing the survey [26] suggest that that individuals may find benefit from social media, as well as encounter feelings of embarrassment, shame, and stigma in their use of social media for sexual and reproductive health information and support.

## Methods

### Overview

This multistage study aims to (1) better understand the relationship between social media and women’s sexual and reproductive health in Aotearoa New Zealand and (2) meaningfully engage with stakeholders and community groups to interpret the findings and propose solutions to identified problems.

This cross-sectional, mixed methods research will be conducted over 24 months in three stages: (1) online data collection, (2) presentation of findings to stakeholders, and (3) pilot focus groups and interviews. This paper addresses the primary stage of data collection (online data collection) and follows the Checklist for Reporting Results of Internet E-Surveys (CHERRIES) checklist [27].

### Survey Design

The survey (Multimedia Appendix 1) targets women living in Aotearoa New Zealand who have used or use social media resources or communities to access information about sexual and reproductive health care. The survey draws from a convenience sample and asks for respondents who have ever identified or currently identify as women, and/or who were assigned female at birth. The survey asks for respondents who are aged 18 years or older, fluent in English, and live anywhere in Aotearoa New Zealand. The survey does not incorporate the ability to check for duplicate responses; nor does it have strict access controls. We recognize that this may impact the veracity of the results as a stand-alone dataset, but as this online survey is intended to be part of a 3-pronged approach collecting multimodal data, we believe that the data collected will be relevant and valuable in conjunction with the focus groups and individual interviews with known, recruited participants.

### Ethical Considerations

#### Institutional Review Board Approval

This survey was approved by the Auckland Health Research Ethics Committee on June 28, 2023, for 3 years. The approval was amended on July 1, 2024, to expand the survey (AH25633).

#### Informed Consent

Participants were provided with a participant information sheet standard to the University of Auckland amended to reflect the specific aims of this research. Participants provided electronic authorization of their consent to participate at the beginning of the survey.

#### Data Protection

Survey responses are held securely at the University of Auckland. Email addresses provided are decoupled from survey responses prior to access by members of the research team. All storage complies with local data security guidelines, and data will be destroyed after 10 years.

#### Compensation

Each focus group and interview participant will receive an NZ $50 (US $28.93) supermarket voucher as *koha* (gift) as a token of acknowledgment for their contribution to the research. Compensation was not provided for completion of the online survey.

### Development and Pretesting

Development and testing of the survey occurred in 2020-2021 as part of a bachelor of nursing (honors) project. This is detailed in the thesis of author HP [26].

### Stage 1: Online Survey

#### Recruitment Process and Description of the Sample Having Access to the Questionnaire (Contact Mode and Advertising the Survey)

**Figure 1:**
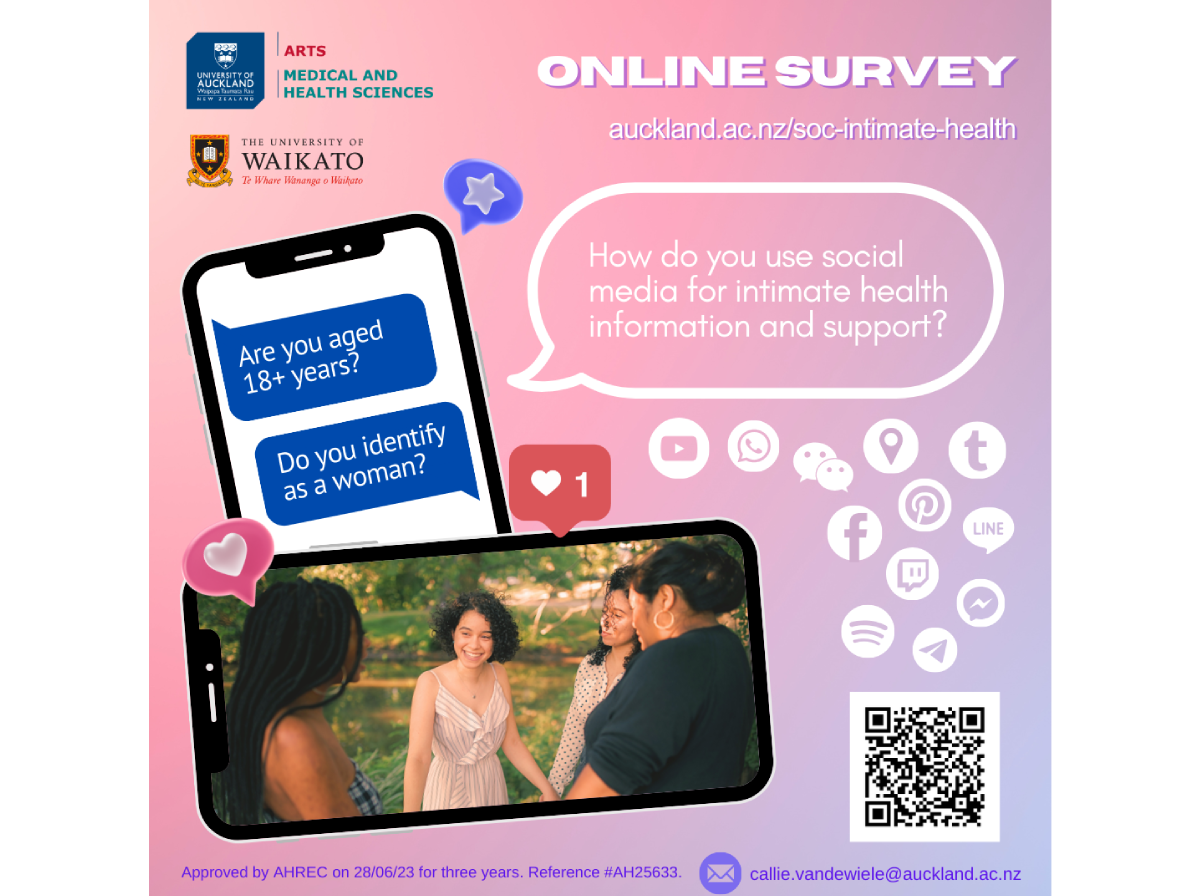
Social media participant recruitment advertisement. The image does not show participants in this study. Image source: Leinio’s Team via Canva.com.

**Figure 2:**
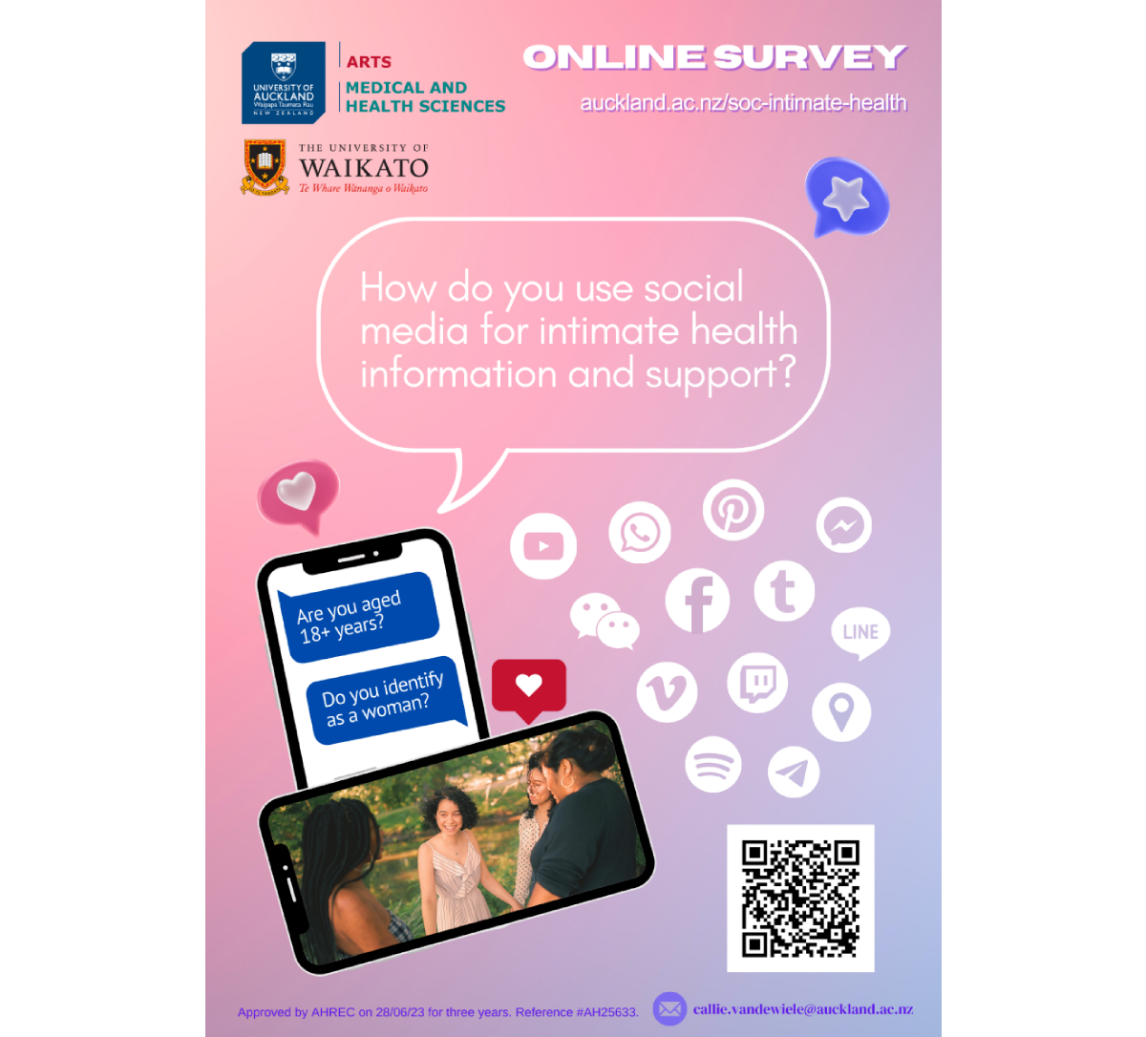
Physical participant recruitment advertisement. The image does not show participants in this study. Image source: Leinio’s Team via Canva.com. Social media logos sourced via images from rawpixel.com on Freepik.

The online survey was an open survey. Participant recruitment occurred through posts from the social media platforms of the Universities of Auckland and Waikato (Figure 1). Funding was allocated to push recruitment among specific population groups that are often underrepresented in online research; these groups are the focus of this study. Physical advertisements of our research were displayed on or in local community notice boards, coffee shops, gyms, libraries, local *marae* (communal and sacred meeting spaces), beauty salons, toilets, and supermarkets nationwide. The physical advertisement is shown in Figure 2. The researchers identified organizations invested in sexual and reproductive health care for sharing digital or poster recruitment flyers. These organizations were recruited as community partners as part of research outreach conducted by members of the research team.

#### Survey Administration

The study used a web survey hosted on a static webpage by the University of Auckland via the Qualtrics XR platform (Qualtrics LLC). The University of Auckland hosts a number of surveys, and each survey includes a standardized information page regarding the research being conducted, as well as links to contact researchers. This research relied on outreach (detailed above in the *Recruitment Process* section) to bring visitors to the University of Auckland survey page. The survey was voluntary and questions were not randomized. The survey was open from July 1, 2024, to February 15, 2025. No monetary incentives were offered for participation in the online survey, but respondents were offered a 1-page summary report of the survey results.

Adaptive questions were used to determine participant eligibility (via self-reporting) but were not used throughout the remainder of the survey. There were a total of 24 questions (including eligibility and consent questions; 19 if these are excluded) across 14 screens. No single screen had more than 4 questions, and questions that were grouped together were related. There was no completeness check conducted for participants before submitting. Respondents were able to reassess questions via the use of a back button.

Regarding the response rate, approximately 500 responses were recorded, of which approximately 320 were complete and indicated that the eligibility requirements were met. Four responses were identified as chatbot responses based on one or more indicative signals (eg, part of the question was included in the answer, which indicates a chatbot response in the experience of the research team, or text from the chatbot tool identifying itself as a chatbot was included). This survey did not control for multiple entries from the same individual at point of collection.

#### Analysis

To prepare the results of the survey for preliminary analysis and stage 2 of the research, an inclusive approach will be used with incomplete survey questions. After informed consent is provided, all responses will be included, even if the entire survey is not complete. This inclusive approach ensures that all available data contribute to the analysis, enhancing the representativeness of participant experiences and reducing response bias. Given the voluntary nature of participation and the potential for survey fatigue or partial completion, excluding incomplete responses could disproportionately exclude certain groups or perspectives. Including partial data supports equity in research by valuing all contributions and allows for more robust descriptive and exploratory analysis, particularly in early-stage or qualitative-informed research.

As we anticipate that only descriptive statistics will be feasible for the survey data, advanced statistical corrections or inferential analyses are not required at this stage. This is appropriate given the exploratory nature of the study and the likelihood of incomplete responses. The primary objective is to identify patterns and trends in the data to inform stage 2 of the research, rather than to draw generalizable conclusions. Descriptive analysis allows for meaningful insights while respecting the limitations of the dataset.

Questionnaires submitted with an atypical timestamp (eg, unusually fast completion times or submissions outside expected timeframes) will be flagged and reviewed for quality assurance. These cases will not be automatically excluded, but rather assessed contextually to determine if the data appear valid and consistent with other responses. This approach ensures that we do not inadvertently exclude legitimate responses from participants who may complete the survey quickly due to familiarity with the content, while still maintaining data integrity.

### Stage 2: Presentation of Findings to Stakeholders

The research team will undertake preliminary data analysis, present these findings to community stakeholders, and engage them for assistance with interpretation and making of meaning. VE, CV, and HP have existing relationships with Māori health care providers, Hāpai Te Hauora, Well Women and Family, Sexual Wellbeing Aotearoa, and the Cancer Society New Zealand, who will all be warmly invited to participate meaningfully. Additional stakeholders may also be invited through the research team and stakeholder networks. It is likely that stage 2 will include a mix of in-person stakeholder *hui* (workshops) and *kanohi ki te kanohi* (face-to-face) meetings individually and in small groups.

*Whakawhanaungatanga* (building relationships) and *manaakitanga* (kindness and respect for others, emphasizing responsibility and reciprocity) will be prioritized throughout, using a flexible approach to best meet the needs, availability, and preferences of the stakeholders. The aim of the stakeholder engagement activities is to obtain feedback on the findings of stage 1 and to facilitate the co-designing of future research priorities. We anticipate that stakeholders will want to co-design in-depth focus groups and interviews with us; we will use the results of these focus groups and interviews as a foundation for further investigation. The focus groups and interviews are described in stage 3.

### Stage 3: Focus Group and Interview Guide and Pilot Focus Groups and Interviews

Based on the results of the survey and the stakeholders’ engagement, a research approach and focus group and interview guide will be developed and piloted with approximately 5 to 8 participants. The authors acknowledge this will require an amendment to existing ethics approval. The aim of stage 3 is not to reach qualitative data saturation but to confirm a research approach and focus group and interview guide for an in-depth qualitative study in the future. Focus groups and interviews will be offered in English and te reo Māori and will be audio-recorded and transcribed verbatim. Focus groups and interviews conducted in te reo Māori will undergo forward and backwards translation into English by a certified University of Auckland or University of Waikato translator. Interview participants will be sent copies of their interview transcript, and they will have 2 weeks to edit or add to the written version of the transcript.

### Data Analysis

The multiple forms of data obtained in stages 1 to 3, including the qualitative survey results, the interpretations and meaning developed with stakeholders, the focus group and interview guide, and the pilot focus group and interview transcripts, will be analyzed together using a collective, team approach to reflexive thematic analysis [28]. Analysis will use NVivo (version 14; Lumivero, LLC). The quantitative survey results will be analyzed using descriptive statistics generated in SPSS (version 30; IBM Corp).

### Results Dissemination

The findings will be disseminated through academic conference presentations, peer-reviewed journal articles, summary reports to participants, detailed reports to stakeholders, and the University of Auckland and University of Waikato social media accounts. As the online portion of the study encompasses 1 of 3 stages, early results will be published with recognition that focus groups and interviews will significantly enhance the understanding of these initial data. Author SM is a medical illustrator and experienced graphic designer who will lead the social media dissemination. We anticipate that this will be completed with significant involvement from stakeholder groups. The results of this survey will be used to prioritize the focus of in-depth qualitative interviews and focus groups in stage 2, detailed above. Additional funding and ethical approval for this future research has been sought.

## Results

Ethical approval for the online survey was granted from the Auckland Health Research Ethics Committee on June 28, 2023, for 3 years, with amendments approved on July 1, 2024 (AH25633). The timeline of our research is presented in Table 1.

**Table 1:** Timeline of our research.

Activity and outcomes			2023		2024				2025		
			October to December		January to June		July to December		January to June		July to December
**Stage 1**											
	AHREC^a^ amendment	✓		✓							
	Extend survey recruitment	✓		✓		✓					
**Stage 2**											
	Stakeholder engagement and *whakawhanaungatanga*^b,c^	✓		✓		✓		✓		✓	
	Analyze preliminary results			✓							
	*Hui* (workshops) with stakeholders			✓		✓				✓^d^	
	Submit papers 1, 2, and 3 for publication					✓					
**Stage 3**											
	Co-design study protocol and external funding applications					✓		✓			
	Apply to the Marsden Fund (NZ $360,000 [US $205,992])					✓		✓			
	Apply to the MBIE^e^ Endeavour Fund (NZ $1 million [US $572,200])							✓			
	Apply for HRC^f^ Project (NZ $1.2 million [US $686,640])									✓	
	Apply for Google Award for Inclusion (NZ $94,000 [US $53,787])							✓			

^a^AHREC: Auckland Health Research Ethics Committee.

^b^*Whakawhanaungatanga*: the process of building and maintaining relationships through shared connections, trust, and mutual respect.

^c^Ongoing throughout.

^d^After submission of funding applications, a celebratory *hui* will be held with all stakeholders and research partners.

^e^MBIE: Ministry of Business, Innovation & Employment.

^f^HRC: Health Research Council of New Zealand.

## Discussion

### Anticipated Findings

The Understanding Intimate Health in Online Spaces project aims to better understand the relationship between social media and women’s sexual and reproductive health in Aotearoa New Zealand. We anticipate (from early results [26]) finding that social media plays a significant role in allowing women to seek out information and care that they would otherwise struggle to access, and that this study can help inform clinical practice in community outreach.

A strength of the approach we have described is the meaningful involvement of stakeholders in the interpretation and meaning-making of the survey findings; in-depth interviews and focus groups provided input into the anticipated next phase of the research. This study takes a multidisciplinary approach bringing together researchers from a variety of disciplines, including public health, biostatistics, nursing, and public policy, as well as anthropology and the social sciences. The aim of doing so is to better understand from diverse perspectives how and if social media impacts some of the most intimate aspects of the health of marginalized groups in Aotearoa New Zealand, namely the sexual and reproductive health of women.

A limitation of our survey is that there were few methods used to assure the validity of participant responses. Responses that were clearly copied and pasted from an artificial intelligence tool were automatically excluded, but no questions were integrated as attention checks.

### Conclusions

The data analyzed to date support the multistage research design, which aims to use this internet survey–based research to launch more targeted in-person and in-community research methods to refine the data collected.

This study was conducted in one particular geographical and sociocultural context (that of Aotearoa New Zealand), and findings may therefore not always be generalizable to other contexts. However, the findings we expect to encounter align with the current literature on social media use and women’s sexual and reproductive health.

The methods used in this study are standard quantitative (survey) and qualitative (survey, focus groups, and interviews) research methods, and can be applied in a variety of contexts. Our study contributes novel insights into the role of social media as a tool for women to access sexual and reproductive health information and support, particularly when these women otherwise have limited access to these resources. Our research also highlights the importance of stakeholder engagement in knowledge production. This protocol provides a framework for investigating this topic in other, international contexts.

## Appendix

Multimedia Appendix 1 Survey.

Multimedia Appendix 2 CHERRIES checklist.

## Abbreviations

LGBTTQIA+: lesbian, gay, bisexual, transgender, takatāpui, queer/questioning, intersex, asexual/aromantic/agender, or another marginalized identity
STI: sexually transmitted infection

## References

[1] Cinelli M, Quattrociocchi W, Galeazzi A, Valensise CM, Brugnoli E, Schmidt AL, Zola P, Zollo F, Scala A (2020). The COVID-19 social media infodemic. Sci Rep.

[2] Tsao S, Chen H, Tisseverasinghe T, Yang Y, Li L, Butt ZA (2021). What social media told us in the time of COVID-19: a scoping review. Lancet Digit Health.

[3] Starrs AM, Ezeh AC, Barker G, Basu A, Bertrand JT, Blum R, Coll-Seck AM, Grover A, Laski L, Roa M, Sathar ZA, Say L, Serour GI, Singh S, Stenberg K, Temmerman M, Biddlecom A, Popinchalk A, Summers C, Ashford LS (2018). Accelerate progress-sexual and reproductive health and rights for all: report of the Guttmacher-Lancet Commission. Lancet Commissions.

[4] Venegas-Vera AV, Colbert GB, Lerma EV (2020). Positive and negative impact of social media in the COVID-19 era. Rev Cardiovasc Med.

[5] Clark JL, Algoe SB, Green MC (2017). Social network sites and well-being: the role of social connection. Curr Dir Psychol Sci.

[6] Marlowe JM, Bartley A, Collins F (2016). Digital belongings: the intersections of social cohesion, connectivity and digital media. Ethnicities.

[7] Giorgio MM, Kantor LM, Levine DS, Arons W (2013). Using chat and text technologies to answer sexual and reproductive health questions: Planned Parenthood pilot study. J Med Internet Res.

[8] Waling A, Farrugia A, Fraser S (2023). Embarrassment, shame, and reassurance: emotion and young people's access to online sexual health information. Sex Res Social Policy.

[9] (2022). "We will buy them back and we will destroy them" - PM Jacinda Ardern on gun control in New Zealand. The Late Show with Stephen Colbert YouTube page.

[10] Braun V (2008). "She'll be right"? National identity explanations for poor sexual health statistics in Aotearoa/New Zealand. Soc Sci Med.

[11] Terry G, Braun V, Farvid P (2012). Structural impediments to sexual health in New Zealand: key informant perspectives. Sex Res Soc Policy.

[12] Rose SB, Garrett SM, McKinlay EM, Morgan SJ (2021). 'Be nice to us, we're still learning': an online survey of young people in Hawkes Bay, New Zealand, about unmet need for sexual health care and improving access to services. Sex Health.

[13] Denison HJ, Bromhead C, Grainger R, Dennison EM, Jutel A (2017). Barriers to sexually transmitted infection testing in New Zealand: a qualitative study. Aust N Z J Public Health.

[14] Came H, McCreanor T, Manson L (2019). Upholding Te Tiriti, ending institutional racism and Crown inaction on health equity. N Z Med J.

[15] Cormack D, Stanley J, Harris R (2018). Multiple forms of discrimination and relationships with health and wellbeing: findings from national cross-sectional surveys in Aotearoa/New Zealand. Int J Equity Health.

[16] Tipene J, Green A (2017). He Pūkenga Kōrero: Rangatahi and sexually transmitted infections in the Waikato. Tewhariki.

[17] Ali A, Scior K, Ratti V, Strydom A, King M, Hassiotis A (2013). Discrimination and other barriers to accessing health care: perspectives of patients with mild and moderate intellectual disability and their carers. PLoS One.

[18] Fredriksen-Goldsen KI, Kim H, Barkan SE, Muraco A, Hoy-Ellis CP (2013). Health disparities among lesbian, gay, and bisexual older adults: results from a population-based study. Am J Public Health.

[19] Harris R, Tobias M, Jeffreys M, Waldegrave K, Karlsen S, Nazroo J (2006). Racism and health: the relationship between experience of racial discrimination and health in New Zealand. Soc Sci Med.

[20] Puhl RM, King KM (2013). Weight discrimination and bullying. Best Pract Res Clin Endocrinol Metab.

[21] Williams DR, Rucker TD (2000). Understanding and addressing racial disparities in health care. Health Care Financ Rev.

[22] Price MJ, Ades AE, Soldan K, Welton NJ, Macleod J, Simms I, DeAngelis D, Turner KM, Horner PJ (2016). The natural history of Chlamydia trachomatis infection in women: a multi-parameter evidence synthesis. Health Technol Assess.

[23] Hider P, Dempster-Rivett K, Williman J, Dempster-Rivett M, Sadler L, McLeod M, Miller A, Sykes P (2018). A review of cervical cancer occurrences in New Zealand 2008-2012. N Z Med J.

[24] Arayasirikul S, Turner C, Trujillo D, Sicro SL, Scheer S, McFarland W, Wilson EC (2022). A global cautionary tale: discrimination and violence against trans women worsen despite investments in public resources and improvements in health insurance access and utilization of health care. Int J Equity Health.

[25] Carastathis A (2014). The concept of intersectionality in feminist theory. Philosophy Compass.

[26] Price H (2021). What are the sexual and reproductive needs of women in Aotearoa New Zealand [honours thesis].

[27] Eysenbach G (2004). Improving the quality of Web surveys: the Checklist for Reporting Results of Internet E-Surveys (CHERRIES). J Med Internet Res.

[28] Braun V, Clarke V (2021). Thematic Analysis: A Practical Guide.

