# Supplementary material for: Understanding Intimate Health in Online Spaces Among Women in Aotearoa New Zealand: Protocol for a Mixed Methods Study
Source: JMIR Res Protoc. 2025 Dec 1;14:e65288. doi: 10.2196/65288 (PMC12706440; doi:10.2196/65288)
Supplement: Multimedia Appendix 1 [file resprot_v14i1e65288_app1.pdf]

Introduction

Introduction

Kia Ora,

You are warmly invited to participate in our anonymous survey about how women use social media for sexual/reproductive health information and support. It will take 10-15 minutes of your time.

We hope to shine a light on how modern-day technology influences women's health and address the taboo topic of sexual and reproductive health. Findings from this study will bring new insight to current research and help inform health promotion policy. Below is a summary of the process for this study.

Full details are in the Participant Information Sheet – Participant Information Sheet PDF

The following topics will be covered:

- How comfortable do you feel accessing information and support online/offline for your sexual/reproductive health?
- How have you used social media to access information or support regarding your sexual/reproductive health?
- What, if any, experiences did you have that led you to seek support and/or information about your sexual/reproductive health?

Eligibility Criteria:

You are invited to take part in this study if you:

- Were assigned female at birth, or if you have ever identified/currently identify as a woman.
- Are aged ≥18 years.
- Currently reside in Aotearoa, New Zealand.
- Are able to understand English, and read and write so that you can complete this survey.

If you have any further questions, please get in

touch with the following:

A.P. Victoria Egli
Division of Health (Nursing), The University of Waikato
Email: victoria.egli@waikato.ac.nz

Dr Callie Vandewiele
Social Sciences (Anthropology), The University of Auckland
Email: callie.vandewiele@auckland.ac.nz

Approved by Auckland Health Research Committee on 15th June 2023 for three years. Amended 1st July 2024. Reference number 25633.

Eligibility

Thank you for showing your interest in this study. We are just going to check if you are eligible to partake:

Were you assigned female at birth, or have you ever identified/do you currently identify as a woman?

- [ ] Yes
- [ ] No

Are you aged 18 years or older?

- [ ] Yes
- [ ] No

Do you live in Aotearoa, New Zealand?

- [ ] Yes
- [ ] No

Can you read and write in English?

- [ ] Yes
- [ ] No

Consent

Consent

You are warmly invited to participate in our survey.

We want to know how women use social media for sexual and reproductive health information/support.

Whether you choose to take part is your choice.

If you do not want to participate or, at any point in the survey, choose to withdraw - you can do so until you "submit" after your final answers. You can do this without having to give a reason.

Please read the attached information below:
Participant Information Sheet PDF

I have read or had someone read the "Participant Information Sheet" to me. I have been given sufficient time to consider whether to participate in this study. I understand that this is voluntary (my choice). I have asked questions if needed and been satisfied with the answers given to me. I know the nature of this research and why I have been invited to participate. I know that my participation in this study is anonymous and that no material which could identify me personally will be used in the reports on this study. I know that once I have submitted my answers to the survey questions, I will not be able to have data removed. I am happy for the research team to collect the data, access, and analyse it.

I agree to take part in this research:

○ Yes
○ No

Survey Questions About Sexual/Reproductive Health

Survey

Do you feel comfortable accessing information or support regarding your sexual/reproductive health in online spaces?

	Not comfortable			Very comfortable	
0	1	2	3	4	5

Slide to answer:

Do you feel comfortable accessing information or support regarding your sexual/reproductive health in offline spaces?

Not comfortable					Very comfortable
0	1	2	3	4	5

Slide to answer: [ ]

Have you ever sought information about your sexual and/or reproductive health from any of the following online sources? Select all that apply.

- [ ] Auckland Sexual Health Service
- [ ] Burnett Foundation Aotearoa
- [ ] Sexual Wellbeing Aotearoa (formerly Family Planning)
- [ ] Fertility NZ
- [ ] Ministry of Health
- [ ] New Zealand Herpes Foundation
- [ ] New Zealand HPV Project
- [ ] New Zealand Sexual Health Society
- [ ] Māori or Iwi Health Providers
- [ ] Pacific Health Providers
- [ ] Migrant Health Providers
- [ ] [ ] Other (please specify)

In what ways have you used social media for sexual/reproductive health support? Select all that apply.

- [ ] Group Chat
- [ ] Private Messaging

- [ ] Interactive Community Pages
- [ ] Voice messaging
- [ ] Video messaging
- [ ] Watching Visual Content
- [ ] Blog Post
- [ ] Other (please specify)
- [ ] I haven't ever used social media for this

On what platforms have you accessed information or support for sexual/reproductive health? Select all that apply.

- [ ] Facebook
- [ ] Facebook Messenger
- [ ] Instagram
- [ ] Twitter
- [ ] Snapchat
- [ ] Tik Tok
- [ ] YouTube
- [ ] Reddit
- [ ] Mastodon
- [ ] Quora
- [ ] Other (please specify)

What do you think are important issues for sexual and reproductive health in Aotearoa, New Zealand?

What is an example of a time when you accessed social media for information and/or support for your sexual/reproductive health?

What, if any, experiences have you had that led you to seek support and/or information about your sexual/reproductive health online?

If you could make sexual/reproductive health information on social media more applicable to people like you, what changes would you make and why?

Is there anything else you want to say about using social media for sexual/reproductive health information and support?

Sarah's Survey Questions

Is how you learn on social media about sexual/reproductive health different from how you learn about other things on social media? If so, how?

What do you think are the benefits of learning about sexual/reproductive health on social media?

What do you think are the risks of learning about sexual/reproductive health on social media? If there are any, what do you do to manage these?

Do you tell anyone else about the information you find when learning about sexual/reproductive health on social media?

Survey Questions About Demographics

How old are you (in years)?

Which ethnic group(s) do you belong to? Select all that apply.

- ☐ Māori

- [ ] Pacific People
- [ ] Asian
- [ ] European
- [ ] NZ European
- [ ] Middle Eastern/Latin American/African
- [ ] ____________ Other
- [ ] I prefer not to say

Where in Aotearoa, New Zealand do you live?

- Auckland
- Canterbury
- Gisborne
- Hawke's Bay
- Manawatu-Wanganui
- Marlborough
- Nelson
- Northland
- Otago
- Southland

Do you live in a:

- ○ City (50,000+ residents)
- ○ Town
- ○ Rural area

Where were you born?

- ○ Aotearoa, New Zealand
- ○ [ ] Outside of Aotearoa, New Zealand. If so, where?

Block 6

End of Survey

Thank you for taking the time to participate in our survey.

It is appreciated. If you have experienced any distress or discomfort when answering these questions, do not hesitate to ask for help. Talk to your trusted support network in the first instance. Other places you can go to for help have been listed below.

If you are distressed, you can contact the following:
Healthline: Phone: 0800 611 116
Lifeline: Phone 0800 543 354 or free text 4357 (HELP)
Women's Refuge: Call their crisis line on 0800 REFUGE (0800 733 843)
Are you affected by sexual harm? Call 0800 044 334 or text 4334 for a free, 24/7, and confidential helpline.

If you have any concerns with your sexual or reproductive health, you can seek out the following:

In the event of an emergency, please call 111.

Call Healthline on 0800 611 116

Call 0800 SEX HEALTH (0800 739 432) to speak with a nurse.

Family Planning: Make an appointment on 0800 372 546.

Find your local New Zealand Sexual Health Clinic here: www.nzshs.org/clinic

Powered by Qualtrics
